# Liquid Chromatography with Tandem Mass Spectrometry: A Sensitive Method for the Determination of Dehydrodiisoeugenol in Rat Cerebral Nuclei

**DOI:** 10.3390/molecules21030321

**Published:** 2016-03-09

**Authors:** You-Bo Zhang, Xin-Bao Yang, Xiu-Wei Yang, Wei Xu, Fei Li, Frank J. Gonzezal

**Affiliations:** 1 State Key Laboratory of Natural and Biomimetic Drugs, Department of Natural Medicines, School of Pharmaceutical Sciences, Peking University Health Science Center, Peking University, No. 38, Xueyuan Road, Haidian District, Beijing 100191, China; zybo5288@163.com (Y.-B.Z.); xbyang0718@163.com (X.-B.Y.); high-xu@163.com (W.X.); 2 Laboratory of Metabolism, Center for Cancer Research, National Cancer Institute, National Institutes of Health, Bethesda, MD 20892, USA; feili2005@gmail.com (F.L.); fjgonz@helix.nih.gov (F.-J.G.)

**Keywords:** HPLC-MS/MS, cerebral nuclei, drug distribution, nutmeg, *Myristica fragrans*, dehydrodiisoeugenol

## Abstract

A new liquid chromatography–tandem mass spectrometry (LC-MS/MS) method is developed for the quantification of dehydrodiisoeugenol (DDIE) in rat cerebral nuclei after single intravenous administration. DDIE and daidzein (internal standard) were separated on a Diamonsil™ ODS C_18_ column with methanol–water containing 0.1% formic acid (81:19, *v*/*v*) as a mobile phase. Detection of DDIE was performed on a positive electrospray ionization source using a triple quadrupole mass spectrometer. DDIE and daidzein were monitored at *m*/*z* 327.2→188.0 and *m*/*z* 255.0→199.2, respectively, in multiple reaction monitoring mode. This method enabled quantification of DDIE in various brain areas, including, cortex, hippocampus, striatum, hypothalamus, cerebellum and brainstem, with high specificity, precision, accuracy, and recovery. The data herein demonstrate that our new LC-MS/MS method is highly sensitive and suitable for monitoring cerebral nuclei distribution of DDIE.

## 1. Introduction

Nutmeg, the seed of *Myristica fragrans* Houtt (family: Myristicaceae) is broadly utilized as a spice and flavoring in African, Asian, and Western sustenance. It has been used subsequent to the seventh century and is known as RouDouKou in Chinese Traditional Medicine [1]. A few studies have uncovered that nutmeg extract has the capacity to stimulate the nervous system [2,3,4] and diminish intestinal tumorigenesis in Apc^min/+^ mice [5]. Dehydrodiisoeugenol (DDIE) is one of the major neolignanoids found in nutmeg [6,7] and the aril [8] of *M. fragrans*. Preliminary work uncovered that DDIE exhibites different bioactivities *in vitro* and *in vivo*, including: antibiotic [8], anti-inflammatory [9], anti-tumor [10], and anti-oxidation [11,12,13]. Nitric oxide (NO) is an endogenous molecule and synthesized from l-arginine by constitutive and inducible nitric oxide synthase (cNOS and iNOS) in numerous mammalian cells and tissues. The excessive production of NO by NOS may be one of the major factors leading to Alzheimer disease, *etc*. [14,15]. In our previous studies, it was found that DDIE extraordinarily restrained the expression of iNOS mRNA [16] and can promptly cross the blood-brain barrier [17,18]. As one of the primary neolignanoids of *M. fragrans*, DDIE is thought to assume a vital part in the sensory system stimulating impacts of nutmeg. Our previous study which utilized high performance liquid chromatography with diode-array detection (HPLC-DAD) reported that DDIE can be distributed into the different zones of the brain [19].

As of late, liquid chromatography–tandem mass spectrometry (LC-MS/MS) innovation has turned into a typical investigative strategy in the pharmaceutical and biomedical fields, especially in bioanalysis. Contrasted with the high performance liquid chromatography ultraviolet (HPLC-UV/DAD) recognition, LC-MS/MS demonstrates high sensitivity and uncommon specificity [20]. In any case, not very many studies have utilized LC-MS/MS to measure drug concentrations in various areas of the cerebrum. Along these lines, the reason for this study is to clear up whether the LC-MS/MS indicates satisfactory accuracy through the investigation of DDIE in rat cerebrum. In the present study, the specificity, accuracy, precision, and recovery of LC-MS/MS, as connected to the quantitation of DDIE in rat cerebrum, was researched. This recently created LC-MS/MS technique was further used to determine the distribution of DDIE in cerebral nuclei in rats.

## 2. Results and Discussion

### 2.1. Chromatographic Conditions

**Figure 1:**
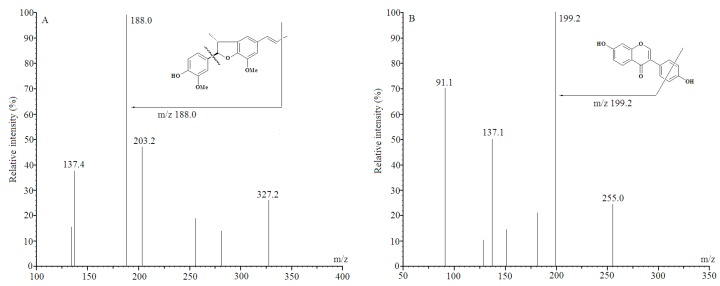
The positive ion scan spectra of DDIE (**A**) and daidzein (I.S.) (**B**).

In this study, the initial step to adding to a technique for DDIE extraction was to optimize the quantity of precursor and product ions of DDIE and internal standard (I.S.) (their chemical structure shown in Figure 1) in multiple reaction monitoring (MRM) mode by syringe pump infusion (flow-rate = 10 μL/min). The standard solutions of DDIE and I.S. were infused into the mass spectrometer independently to acquire detected ions and to optimize mass parameters, for example, declustering potential (DP) and collision energy (CE), and after that positive mode (ESI+) was utilized to enhance sensitivity, reproducibility and fragmentation of the analyte. The full-scan turboionspray product ion mass spectra identified in positive ion mode demonstrated that the precursor ions of DDIE and I.S. were the protonated particles with [M + H]^+^ of *m*/*z* 327.2 and *m*/*z* 255.0, individually. After collision-induced dissociation, the most copious particles in item particle mass range were at *m*/*z* 327.2→188.0 for DDIE, with a CE of 29.0 eV, and at *m*/*z* 255.0→199.2 for I.S., with CE of 37.0 eV, for the identification and measurement of DDIE in MRM mode.

**Figure 2:**
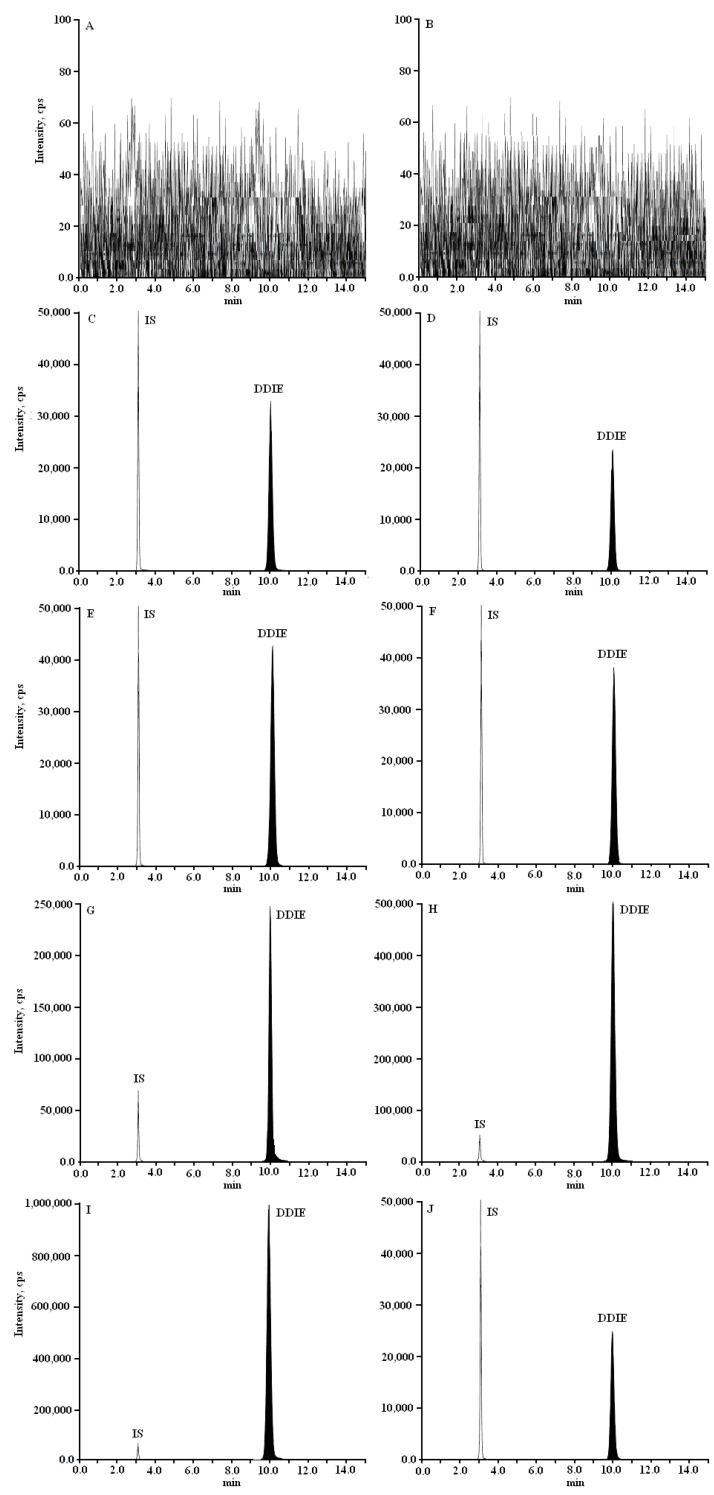
Representative MRM chromatograms of DDIE (*m*/*z* 327.2/188.0) and daidzein (I.S.) (*m*/*z* 255.0/199.2) in rat plasma and cerebral nuclei samples after intravenous administration of DDIE at a single dose of 40 mg/kg to rats: (**A**) blank plasma; (**B**) blank hippocampus; (**C**) blank hippocampus spiked with DDIE and I.S.; (**D**) hippocampus; (**E**) striatum; (**F**) hypothalamus; (**G**) cerebellum; (**H**) brainstem; (**I**) cortex; (**J**) plasma.

The DDIE and I.S. were separated successfully on a Diamonsil™ ODS C_18_ column without interference. The methanol (MeOH)–water (H_2_O) containing 0.1% formic acid (81:19, *v*/*v*) system was determined to be the mobile phase at flow-rate of 1.0 mL/min. Formic acid was added to improve the peak shape and to increase sensitivity. The typical chromatograms of DDIE and I.S. in rat plasma and cerebral nuclei are shown in Figure 2.

### 2.2. Specificity

The LC-MS/MS chromatograms of rat plasma and cerebral nuclei samples are shown in Figure 2. Plasma and cerebral nuclei samples showed no interfering peaks from endogenous substances. Retention times for DDIE and I.S. were 10.0 and 3.1 min, respectively.

### 2.3. Linearity of Calibration Curves and Lower Limits of Detection and Quantification

As summarized in Table 1, all of the correlation coefficients (*r*^2^) were ≥0.9908 indicating good linearity of calibration curves in each of the concentration ranges. The lower limit of detection (LLOD) of 3.2 ng/mL and the lower limit of quantification (LLOQ) of 12.0 ng/mL were determined as the suitable thresholds for quantitation of DDIE, which are sufficient to support the study of this molecule’s distribution in cerebral nuclei.

### 2.4. Precision and Accuracy

Quality control (QC) samples at low, medium, and high concentrations were analyzed for determining the accuracy and precision of HPLC-MS/MS. For intra-day precision, the relative standard deviations (RSDs) of all QC samples were less then 14.5% with intra-day accuracies ranging from 88.8% to 112.9%. The inter-day precisions (RSDs) for all QC samples were less than 10.0% and the inter-day accuracies ranged from 90.0% to 114.1% (Table 2).

### 2.5. Absolute Recovery and Stability

The absolute recovery of DDIE was estimated at three concentrations and calculated by comparing the peak areas from plasma and cerebral nuclei tissue samples to those of the peak areas from pure DDIE in MeOH at the same concentration. The mean recoveries of DDIE in the plasma and tissue samples ranged from 78.4% to 89.2% (Table 2). DDIE was found to be stable in the plasma and tissue samples after three freeze–thaw cycles with no significant degradation observed (Table 2).

### 2.6. Matrix Effects

Matrix interferences were evaluated by analyzing the peak areas of the mobile phase and post-extraction blank samples, both were mixed with three QC concentrations of DDIE and 50 μg/mL of I.S. with the same concentrations. As shown in Table 2, the ratios of DDIE ranged from 86.6% to 97.4%, suggesting that no matrix effects were present at the retention time of the analytes. The ratios of I.S. were in the range of 89.5% to 98.1%.

### 2.7. Application to Cerebral Nuclei Distribution Study

**Figure 3:**
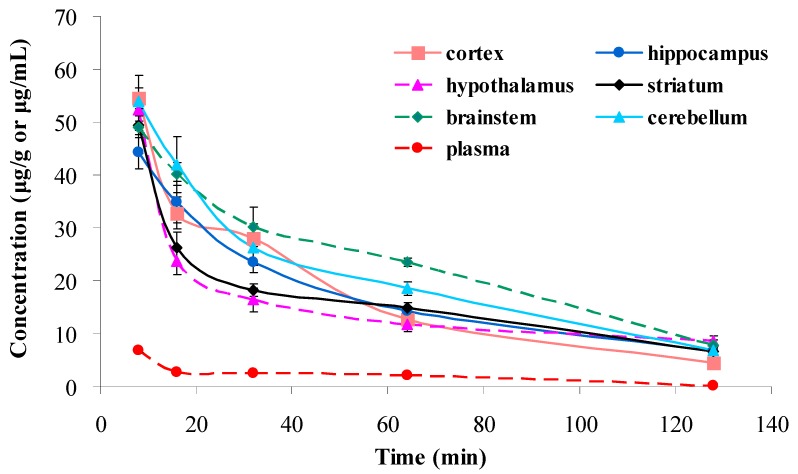
Concentration-time profiles of DDIE in cerebral nuclei and plasma after intravenous administration of DDIE at a single dose of 40 mg/kg to rats (*n* = 5). The units of DDIE in cerebral nuclei and plasma are μg/g and μg/mL, respectively.

The newly developed LC-MS/MS method was successfully applied to the determination of DDIE in rat cerebral nuclei after intravenous administration of DDIE (Figure 3). Results from the current study indicated that DDIE had a rapid distribution to various cerebral nuclei, and it can be detected in all of the assayed cerebral nuclei between 8 and 128 min. As seen in Table 3, DDIE showed extensive distribution characteristics. After intravenous administration, the concentration of DDIE in cerebral nuclei showed dynamic changes and then gradually reduced over time, similar to the dissipation rate of DDIE in the plasma.

A sensitive and reproducible method using LC-ESI-MS/MS to quantify DDIE in rat cerebral nuclei is developed and implemented in this study. LC-ESI-MS/MS is a hyphenated mass spectrometry technique that combines the separation capability of HPLC and the high mass accuracy of a mass spectrometer. Recent developments in MS technology have greatly improved the specificity and sensitivity of this technique. A triple quadrupole instrument has been widely used for biological sample analysis. Using daidzein as an internal standard, the current study has significantly increased the sensitivity of the methodology compared with previous DDIE research *in vivo* [17,21,22]. Our previous study reported that DDIE can be distributed into the brain through the HPLC-DAD method [19]. The current study demonstrated that the LC-MS/MS method showed satisfactory specificity, precision, accuracy, and recovery for the quantification of DDIE concentration in cortex, hippocampus, striatum, hypothalamus, cerebellum, and brainstem. These results indicated that the concentrations of DDIE in cerebral nuclei using LC-MS/MS method were very similar to its concentration in cerebral nuclei quantified by HPLC-DAD [19], suggesting that the LC-MS/MS analysis is a reliable method for the determination of drug distribution in brain nuclei. More importantly, the LC-MS/MS method for detection of DDIE in brain nuclei showed more sensitivity than HPLC-DAD. In the present study, the LLOD and LLOQ of LC-MS/MS were 3.2 ng/mL and 12.0 ng/mL, which were significantly lower than detectable limits by HPLC-DAD, which are 10 ng/mL and 40 ng/mL, respectively [19]. Therefore, LC-MS/MS technology is highly suitable for the analysis of drug concentrations in cerebral nuclei.

## 3. Experimental Section

### 3.1. General Information

DDIE was separated from nutmeg with purity >99.5% as described previously [7], and its structure was confirmed by MS, ^1^H- and ^13^C-NMR spectral analyses. Daidzein (internal standard, purity >99%) was purchased from National Institutes for Food and Drug Control (Batch No.111502-200101, Beijing, China). HPLC/MS-grade MeOH was purchased from J. T. Baker (Center Valley, PA, USA). HPLC-grade formic acid was obtained from Dikma Tech. Inc. (Beijing, China). H_2_O was collected from a Milli–Q Ultra–pure water system (Millipore, Billerica, MA, USA).

### 3.2. Instrumentation and Conditions

The analytical Dionex Ultimate 3000 HPLC system (Dionex Corp., Sunnyvale, CA, USA) consisted of an Ultimate 3000 Pump, a Dionex Ultimate 3000 Autosampler and a DIONEX Ultimate 3000 Compartment. The Applied Biosystems 4000QTRAP triple quadrupole tandem mass spectrometer (Applied Biosystems Inc., Toronto, ON, Canada) was equipped with an electrospray ionization (ESI) source for the mass analysis and detection. All data collected were analyzed and processed using the Analyst 1.5.1 software (Applied Biosystems Inc.). The analysis was performed on a Diamonsil™ ODS C_18_ column (250 × 4.6 mm i.d., 5 μm; Dikma, Beijing, China) pre-equipped with a C_18_ guard column (8 × 4 mm i.d., 5 μm, Dikma, Beijing, China). The mobile phase was MeOH-H_2_O containing 0.1% formic acid in a ratio of 81:19 (*v*/*v*) with flow-rate of 1 mL/min and injection volume was 5 µL. The Turbo ionspray source was set in positive ionization mode. MRM was used to detect sequence-specific transitions at *m*/*z* 327.2→188.0 for DDIE and *m*/*z* 255.0→199.2 for I.S. The ion spray voltage was set at 5500 V and the source temperature was set at 550°C. The collision activated dissociation (CAD) was set at 2.0 utilizing nitrogen as collision gas. Nitrogen was also used as curtain gas, nebulizing gas, and heater gas with pressures of 15, 60, and 50 psi, respectively.

### 3.3. Animals

Male Sprague-Dawley (SD) rats weighting 190–210 g were housed and bred in the Laboratory Animal Center of Peking University Health Science Center (Beijing, China). The rats were maintained in a 12 h light/12 h dark cycle and temperature-controlled (22 ± 1 °C) environment, and allowed free access to standard feedstuff and water until 12 h before the experiment, at which time food was removed. All procedures were conducted in accordance with the Guide for the Care and Use of Laboratory Animals published by AAALAC and approved by the Peking University Health Science Center Committee on Animal Care and Use (SYXK [Jing] 2006-0025; No. LA2014162).

### 3.4. Preparation of Standard and Quality Control Samples

The working solution of DDIE was prepared from the stock solutions (1.0 mg/mL in MeOH) and was thawed at room temperature prior to use. The I.S. working solution was prepared by diluting the I.S. stock solution (1.0 mg/mL) with MeOH to obtain the concentration of 50 μg/mL. The cerebral nuclei and plasma calibration standards of DDIE were prepared by further spiking drug-free rat cerebral nuclei and plasma samples with 20 μL of I.S. and appropriate DDIE working solutions to construct the calibration curves. QC samples were prepared in a similar manner to give three levels of low, medium and high concentrations.

### 3.5. Sample Preparation

Liquid-liquid extraction of plasma samples (200 μL) was performed by adding 20 μL of I.S. working solution and 800 μL of ethyl acetate (EtOAc). Samples were vortexed for 1 min, and then centrifuged at 16,000 *g* for 10 min. The upper organic phase was transferred into a 5 mL glass tube and the remnant layer was extracted with EtOAc again as described above. The supernatants from both extracts were combined and evaporated to dryness with a gentle stream of nitrogen at 40 °C. The residues were reconstituted in 1 mL of MeOH and a 5 μL aliquot solution was injected into the LC-MS/MS system for analysis. The cerebral nuclei samples were thawed at room temperature and homogenized in 1 mL of pre-cooled 0.9% saline. One mL of the tissue homogenates, 4 mL of EtOAc, and 20 μL of I.S. working solution were combined and mixed for 1 min and then were processed in the same manner as the plasma samples at the beginning of Section 2.5. The residue was dissolved in 1 mL of MeOH (for cortex samples, the residue was reconstituted in 3 mL of MeOH) and 5 μL of each solution was injected into the HPLC-MS/MS system for analysis.

### 3.6. Method Validation

To evaluate the validation of HPLC-MS/MS method, the specificity, linearity, precision, accuracy, stability, absolute recovery, and matrix effects were investigated. Specificity of detection of DDIE against endogenous interferences was assessed by injecting blank blood samples and blank cerebral nuclei samples into the HPLC-MS/MS system to exclude the interference from endogenous metabolites. Calibration curves were constructed using DDIE/I.S. peak area ratios versus DDIE concentration, and each calibration curve consisted of six concentration levels from both cerebral nuclei and plasma samples. The LLOQ and the LLOD were estimated as the lowest concentrations of DDIE resulting in signal-to-noisy ratios (S/N) of 10:1 and 3:1, respectively.

The intra- and inter-day precisions were expressed as the RSDs and the accuracy was calculated by comparing the calculated concentration with the original starting concentration. Three concentrations of QC samples were assayed five times on the same day to evaluate the intra-day precision and accuracy, and were analyzed each on three consecutive days to determine the inter-day precision and accuracy. The absolute recovery of plasma and cerebral nuclei samples was evaluated by comparing the analytical results of extracted samples at three QC concentrations with pure standards in MeOH at the same concentration. The matrix effects on ionization were measured by comparing the peak areas of DDIE and I.S. dissolved in the pre-extracted blank plasma and cerebral nuclei samples with that of the pure standard solutions containing equivalent amounts of the analytes.

The stability of DDIE in rat cerebral nuclei homogenate and plasma was evaluated. The blank plasma and cerebral nuclei samples that were spiked with DDIE and I.S. were stored at −20 °C for 24 h, and then were thawed at room temperature for 12 h. This cycle was repeated three times and analysis was performed after the third cycle. The conditions for stability analysis adequately covered the storage conditions of the research samples.

### 3.7. Distribution Study

Thirty male rats were used in this study and randomly divided into six groups of five each, with one group assigned as the control group. The five experimental groups were administered 40 mg/kg DDIE intravenously into the tail vein using a syringe. Approximately 1.0 mL blood samples were withdrawn in clean heparinized centrifuge tubes through intraorbital puncture at 8, 16, 32, 64, and 128 min. After perfusion with 0.9% physiological saline, the rat brain was removed at the five time intervals mentioned above. The six cerebral nuclei, cortex, hippocampus, striatum, hypothalamus, cerebellum, and brainstem were quickly stripped and washed with 0.9% saline solution. All samples were weighed and stored at −20 °C prior to use.

### 3.8. Statistical Analysis

Statistical analysis was performed on Microsoft Excel 2003 (Microsoft Corp., Seattle, WA, USA) and pharmacokinetic parameters were calculated by DAS 2.0 software (Drug and Statistics 2.0, Mathematical Pharmacology Professional Committee of China, Shanghai, China).

## 4. Conclusions

A rapid, simple and sensitive LC-MS/MS method for determination of DDIE in rat cerebral nuclei has been developed for the first time. The determination of drug distribution in brain nucleus tissue is difficult because of the small amount of the brain nuclei tissue and the drug blood-brain barrier mechanism. The developed method showed good specificity, precision, accuracy, and recovery, and displays superior sensitivity and specificity to the HPLC-DAD detection method. It can significantly lower the detective and quantitative limits of DDIE in samples. The current results indicated that DDIE can rapidly cross the blood brain barrier and exhibited similar pharmacokinetic action as in plasma. This study also suggested that DDIE might exert pharmacological action on the central nervous system *in vivo* and might be one of the main bioactive components of nutmeg. The data presented herein demonstrated the power of LC-MS/MS to quantify drug concentrations in cerebral nuclei following drug treatment.

## Figures and Tables

**Table 1:** Calibration curve, correlation coefficient (*r*^2^) and linear range of DDIE in rat plasma and cerebral nucleus samples.

Sample	Calibration Curve	*r*^2^	Linear Range (μg/mL)
Plasma	*y* = 1.5501*x* + 0.0719	0.9940	0.05–2.0
Hippocampus	*y* = 1.2178*x* + 0.0420	0.9948	0.05–1.0
Striatum	*y* = 2.1454*x* − 0.1719	0.9956	0.10–2.0
Cortex	*y* = 1.7553*x* + 0.1834	0.9908	2.00–32
Cerebellum	*y* = 1.5364*x* + 0.0358	0.9972	1.00–20
Brainstem	*y* = 1.5864*x* + 0.3528	0.9944	0.50–12
Hypothalamus	*y* = 1.6004*x* + 0.0800	0.9948	0.05–1.5

**Table 2:** Extraction recovery, stability, matrix effect, intra- and inter-day accuracy, and precision of DDIE in rat plasma and cerebral nuclei samples.

Samples	Spiked (μg/mL)	Intra-Day			Inter-Day			Recovery		Stability		Matrix Effect	
		Measured (μg/mL)	RSD (%)	Accuracy (%)	Measured (μg/mL)	RSD (%)	Accuracy (%)	Mean (%)	RSD (%)	Accuracy (%)	RSD (%)	Mean (%)	RSD (%)
Plasma	0.25	0.24	7.31	96.81	0.25	3.14	99.10	84.31	1.91	94.77	5.58	93.02	7.22
	0.50	0.55	6.86	110.5	0.57	2.46	113.3	81.41	1.60	108.7	5.47	95.02	7.76
	1.50	1.55	5.08	103.6	1.57	1.42	104.4	86.01	3.60	103.8	5.06	95.10	2.62
Hippocampus	0.10	0.09	14.5	94.76	0.11	9.37	105.4	84.09	3.70	94.40	13.9	90.26	4.90
	0.20	021	7.54	104.6	0.22	2.02	111.3	79.83	4.80	110.4	3.41	94.58	11.9
	0.80	0.80	3.77	100.4	0.79	5.19	98.20	80.35	1.17	97.73	2.64	96.10	10.7
Striatum	0.25	0.27	3.40	107.1	0.27	4.61	107.4	82.33	13.6	107.8	2.46	93.09	9.44
	0.50	0.44	4.72	88.84	0.45	5.44	89.98	88.72	9.99	85.85	2.37	90.49	7.28
	1.50	1.38	1.48	92.33	1.43	2.48	95.66	87.82	7.63	93.91	4.02	90.33	5.18
Cortex	4.00	3.98	2.22	99.42	3.99	2.61	99.67	83.97	7.98	99.61	2.04	91.78	2.53
	8.00	7.49	7.12	93.64	7.79	3.25	97.31	87.52	4.42	94.36	2.11	97.44	1.41
	24.0	23.1	9.50	96.06	22.0	4.77	91.74	83.29	8.47	92.20	4.55	95.16	5.20
Cerebellum	2.50	2.61	7.45	104.4	2.69	2.64	107.7	84.35	7.62	104.5	7.55	90.42	5.33
	5.00	5.43	4.87	108.7	5.26	5.53	105.2	81.37	9.14	108.1	5.80	91.74	8.46
	15.0	15.2	5.20	101.2	15.4	3.93	102.7	87.37	1.42	99.84	3.29	92.22	4.31
Brainstem	1.00	0.97	6.77	96.95	0.94	9.29	93.94	82.67	8.57	89.61	1.51	93.59	2.79
	2.00	1.85	6.74	92.50	1.81	4.83	90.71	85.41	2.94	89.48	5.36	86.58	3.59
	8.00	8.33	2.47	104.2	8.13	4.13	101.6	81.75	5.45	100.4	3.15	94.39	10.3
Hypothalamus	0.10	0.11	2.52	112.9	0.11	2.62	114.1	78.42	3.10	112.6	2.14	94.20	6.87
	0.30	0.30	13.0	101.0	0.33	2.10	109.9	89.20	6.17	99.80	11.7	90.28	5.09
	1.20	1.25	5.28	103.9	1.27	1.69	105.5	82.33	3.50	103.2	4.70	93.58	4.64

**Table 3:** Pharmacokinetic parameters of DDIE in rats plasma and cerebral nuclei after *i.v.* administration at a single dose of 40 mg/kg (mean ± SD, *n* = 5).

Samples	t_1/2_ (min)	AUC_0→t_ (μg·min/g)	AUC_0→∞_ (μg·min/g)	CL (μg·kg/min)	MRT (min)	C_max_ (μg/g)	V_1_ (L/kg)
Plasma	54.326 ± 0.877	309.679 ± 13.254 ^a^	479.692 ± 26.388 ^a^	0.083 ± 0.005 ^b^	42.709 ±1.701	6.827 ± 0.278 ^c^	0.008 ± 0.005
Hippocampus	62.212 ± 3.833	2631.272 ± 230.935	2463.297 ± 300.575	0.012 ± 0.001	63.460 ±0.751	44.406 ± 3.236	0.669 ± 0.025
Striatum	69.315 ± 0.010	2498.242 ± 226.990	3542.121 ± 327.205	0.011 ± 0.001	73.439 ± 0.330	49.506 ± 5.411	0.213 ± 0.022
Cortex	38.227 ± 2.283	2742.581 ± 299.720	3400.941 ± 429.470	0.012 ± 0.002	46.490 ± 3.270	54.539 ± 4.363	0.001 ± 0.001
Cerebellum	56.059 ± 1.272	3144.154 ± 250.522	4083.262 ± 251.445	0.010 ± 0.001	57.935 ± 841.000	53.940 ± 1.391	0.504 ± 0.064
Brainstem	55.923 ± 2.433	3372.296 ± 223.017	4447.434 ± 297.052	0.009 ± 0.001	65.185 ± 2.772	49.082 ± 2.120	0.372 ± 0.312
Hypothalamus	69.315 ± 0.001	2418.876 ± 241.893	3887.033 ± 222.825	0.011 ± 0.001	116.471 ± 27.269	52.423 ± 3.986	0.199 ± 0.008

The units of a, b and c were μg·min/L, L·kg/min and μg/L, respectively; V_1_, the central distribution volume.
